# Case of Menstruation in the Male

**Published:** 1867-03

**Authors:** V. O. King


From the So. Jour. of Medical Sciences.

### *Case of Menstruation in the Male.*

Messrs. Editors.—Permit me gentlemen, through the medium of your Journal, to lay before the profession the particulars of an anomaly, which will doubtless prove interesting to your readers.

In the month of December, 1855, while attending the medical lectures of the University of Louisiana, I formed the acquaintance of a young gentleman, who was then a student in the same school. His character and attainments were such as to commend themselves to my admiration, which subsequently ripened into friendship and intimacy.

On one occasion, in a moment of extreme melancholy, and under the influence of the confidential relations existing between us, he communicated to me, that though not possessed of the usual organs, he periodically performed the simulated functions of menstruation; and that this deviation from the laws of nature, in his person, was not only inexplicable, but the source of the most painful and gloomy reflections. I enjoyed peculiar opportunities of observation, which were faithfully improved. He had been the victim of this vicarious function for a period of three years, eliminating an apparent catamenial secretion, with the same regularity, and attended by the same indications by which it is characterized in the human female.

The fluid exuded, flowed from the sebaceous glands of the deep fossa behind the corona glandis, and was of a sanguineous appearance, homogeneous and thick. The quantity of this exudation varied from one to two ounces during each hemorrhagic period, and the duration of the periods from three to six days. The subject was then about twenty-two years of age, of a lymphatic temperament, and had never been contaminated by venereal disease. Though not prone to the indulgence of lustful passions, he was not innocent of having sometimes yielded to their promptings, which were especially potent immediately preceding his periodical purifications. I was made the sole repository of this gentleman's gloomy secret, and have not been heretofore permitted to divulge it; and even now the injunction of silence is but partly removed.

Medical literature abounds with the recital of strange and unaccountable departures from the boundaries, to which Nature has restricted this and kindred functions. The mammary gland in man, though only rudimentary in structure and conformation, has been known to rival its congener in woman, developing lobes, vesicles, excretory ducts, and areolar tissue. Its lactiferous function has been perfected, under certain auspices, to such a degree as to yield its peculiar nourishment for an indefinite time. Women have been known to menstruate through unusual channels, and at unusual times—the anus, the mouth, the nose, the ears, ulcerated surfaces, and the very pores of the skin have been made tributary to this sexual function. Females have menstruated during pregnancy, during lactation, in old age, and even in the tender years of infancy; while others, in the vigor of life and in robust health have never menstruated at all.

But this case, so analagous in all its features to the catamenial phenomenon, has no parallel that has fallen under my notice, and I believe stands alone in point of its wide divergeance from Nature.

Hoping that the subject matter of this paper may awaken inquiry, and perhaps arouse intelligent discussion, or that it may elicit the publication of similar phenomena, I have the honor to be, very respectfully, V. O. King, M. D.

